# Children and youth’s movement behaviours differed across phases and by geographic region throughout the COVID-19 pandemic in Nova Scotia, Canada: an explanatory sequential mixed-methods study

**DOI:** 10.1186/s44167-023-00032-6

**Published:** 2023-11-03

**Authors:** Julie E. Campbell, Michelle R. Stone, Raktim Mitra, Maggie Locke, Cynthia MacDonald, Ashley Preston, Rebecca A. Feicht, Laurene Rehman, Sara F. L. Kirk, Guy Faulkner, Mark S. Tremblay, Sarah A. Moore

**Affiliations:** 1 Faculty of Health, School of Health and Human Performance, Dalhousie University, PO Box 15000, Halifax, NS B3H 4R2 Canada; 2 Healthy Populations Institute, Dalhousie University, PO Box 150000, Halifax, NS B3H 4R2 Canada; 3 School of Urban and Regional Planning, Toronto Metropolitan University, 105 Bond Street, 4th Floor, Toronto, ON M5B 1Y3 Canada; 4 School of Kinesiology, University of British Columbia, 6081 University Blvd, Vancouver, BC V6T 1Z1 Canada; 5 Children’s Hospital of Eastern Ontario Research Institute, 401 Smyth Road, Ottawa, ON K1H 8L1 Canada; 6 Department of Pediatrics, University of Ottawa, 401 Smyth Road, Ottawa, ON K1H 8L1 Canada; 7 Department of Pediatrics, Faculty of Medicine, Dalhousie University, PO Box 15000, Halifax, NS B3H 4R2 Canada

**Keywords:** Children and youth, Physical activity, Sedentary behaviour, Sleep, Environment, Public health restrictions

## Abstract

**Background:**

Like many places globally, the health and well-being of children and youth living in Canada were significantly affected by the COVID-19 pandemic. Restricted access to the outdoors, schools, and public green spaces impacted children’s physical activity (PA), sedentary behaviour, and sleep. Restrictions changed throughout the pandemic, and children’s and youth’s movement behaviours may have been differentially affected based on time and place. This paper aimed to examine the impact of the COVID-19 pandemic on the movement behaviours of children and youth living in Nova Scotia (NS), Canada, over time and by geographic region using mixed methods.

**Methods:**

This study employed an explanatory sequential mixed-methods design. Secondary data from three repeated cross-sectional surveys of parent-reported demographic, movement, and geographic data of 291 children and youth aged 5–17 years were analyzed. Spatial cluster analyses were applied to identify geographic concentrations of children and youth who were more or less likely to meet the moderate-vigorous PA (MVPA) guideline during the pandemic. Semi-structured interviews were conducted with 14 Nova Scotian parents to understand their perspectives on their child’s movement behaviours during the pandemic. Interviews were analyzed deductively based on quantitative results using reflexive thematic analysis.

**Results:**

Our findings showed only 5.5% of children and youth were meeting all guidelines throughout the pandemic. Of the movement behaviours, screen time (ST) differed across the pandemic and by age and gender. Clusters of children and youth meeting the MVPA recommendation on fewer days were found in regions within NS’s three largest population centres (Truro, Sydney, and the Halifax Regional Municipality (HRM)), and clusters of those meeting the MVPA recommendation on more days were also identified in the HRM. From semi-structured interviews, themes indicated: (1) escaping screens during early parts of the COVID-19 pandemic and when weather was colder was hard; (2) having access to spaces to be active near the home helped facilitate children’s movement; and (3) higher socioeconomic status enabled more opportunities for movement.

**Conclusion:**

Overall, fewer public health restrictions led to more favourable movement behaviours and spatial and sociodemographic factors may have been at play. Decision-makers should consider these factors when identifying strategies to keep children active during future health crises.

**Supplementary Information:**

The online version contains supplementary material available at 10.1186/s44167-023-00032-6.

## Background

The health and well-being of Canadian children and youth have been significantly affected by the COVID-19 pandemic and related public health measures. Children and youth were less active and spent more time being sedentary (particularly on screens), engagement in outdoor time and play declined, and mental health and well-being were negatively impacted [1–9]. Restricted access to the outdoors (including playgrounds and public green spaces), closures of schools and childcare settings, and cessation of sports and recreation activities reduced outdoor play and movement opportunities; this warrants concern as play and movement are critical for optimizing and maintaining physical, cognitive, social, and emotional health [10, 11]. In Canada in the first months of the pandemic, just 4.8% of children and 0.6% of youth met the combined 24-h movement behaviour guidelines (physical activity (PA), screen time (ST), sleep [12]) [6]; guideline adherence was still low six months later (4.5% of children and 1.9% of youth) [7], and these figures were drastically lower than the pre-pandemic levels, where 15% of 5–17-year-olds were meeting guidelines as per the Canadian Health Measures Survey [13]. These trends were similarly experienced in countries worldwide at this time [14].

The socioecological model (SEM) recognizes that health behaviours are determined by much more than just the individual’s choices, with many levels interacting to influence health [15–17]. Built- and social neighbourhood characteristics are levels that can influence children and youth’s movement behaviours [18–20]. Recent research of Canadian neighbourhood environments during the COVID-19 pandemic demonstrated that low density dwellings, and access to parks in high density neighbourhoods improved the odds of increased outdoor activities during the pandemic, while proximity to major roads was a barrier [5]. Social conditions in a neighbourhood, such as housing insecurity, ethnic diversity, and unemployment levels, have also been found to be related to children and youth’s movement at different stages of the pandemic [21]. Another level of the SEM critically related to children and youth’s movement during the pandemic is the outer policy level that governs restrictions and guidelines put in place to mitigate virus transmission.

Given that restrictions changed throughout the pandemic based on case counts in certain areas of the world and country, children’s and youth’s movement behaviours may have been differentially affected based on time and place. To date, Canada-wide and regional analyses of movement behaviour guideline adherence during the early stages (spring of 2020 and fall of 2020) of the pandemic have been conducted (e.g., [1, 3, 6, 7]). However, the Atlantic Region of Canada (New Brunswick, Nova Scotia, Prince Edward Island, and Newfoundland and Labrador) was grouped in those analyses [1, 3]. Atlantic Canada had fewer cases of COVID-19, fewer restrictions to the outdoors, and less severe declines in children’s and youth’s outdoor time and play during the pandemic compared to other regions in Canada [3]. Additionally, no analyses have been conducted in this region using data from the third data collection period (spring 2021) during the pandemic.

Nova Scotia also provides a unique case study due to it being the region’s most populated (~ 1 million residents) and culturally diverse population. Additionally, its advantageous geography of being a maritime province, relatively separate from other provinces and territories, allowed Nova Scotia to have control over its entry points to implement strict boarder controls and limit the introduction of new COVID-19 cases from outside the area. The surrounding Atlantic provinces were also entered into an agreement with Nova Scotia known as the “Atlantic Bubble” from June 2020 until April 2021, allowing residents to travel freely within the region without quarantine requirements. Internationally, this is comparable to other regions or countries who implemented similar practices, such as fully-closed borders or travel zones, facilitating low transmission, safe travel, and economic benefits, including the Australia/New Zealand Travel Bubble the Baltic Travel Bubble of Estonia, Latvia, and Lithuania. Thus, potentially contributing to evidence on these models in the mitigation of virus transmission while also preserving access to areas and amenities related to favourable movement behaviours. Given this context, there is value for a focused case-study analysis of the pandemic-related differences in movement behaviour guideline adherence of the province of Nova Scotia, at various time points, and across different geographic locations, of the first year of the COVID-19 pandemic.

To provide some local context of the COVID-19 pandemic in Nova Scotia, the first wave of the pandemic in spring 2020 coincided with the first data collection period of this study’s survey (April 2020). At this time in Nova Scotia, the province had declared a state of emergency; most activities were cancelled, schooling was online, recreation areas (parks, playgrounds, sports facilities, trails, campgrounds, community gardens) were closed, and gatherings were limited to those in one’s household [22]. Case trends and restrictions were comparable to countries such as Australia and New Zealand, which were low compared to Canada as a whole [23]. Throughout the late spring, summer, and early fall, case counts dropped significantly, allowing recreation areas and facilities and gathering limits to be re-opened or expanded. During the second data collection, six months later, in October 2020, contrary to other regions in Canada, case counts in Nova Scotia were relatively low [24]. A state of emergency was renewed mid-October as a preventative measure, with cases rising in other areas of the country [25] and worldwide [23]. Restrictions continued to be fairly limited until late-November of 2020, when cases started to rise again, events and gatherings were not permitted, and varying restrictions continued into the winter months [26]. Finally, when the third iteration of the survey was conducted in April of 2021, the third wave of the pandemic was upon Nova Scotia [24], and schools and other activities were closed again [27]. Cases started to come back down in May but remained higher than in other periods of the pandemic throughout June and July of 2021 [24], which corresponded to the timing of the parental interviews for this current study. Of note, in May 2023, COVID-19 has been downgraded and no longer constitutes a public health emergency of international concern, though experts continue to suggest COVID-19 in Canada is still a highly endemic infectious disease [28].

Given the varying case counts and related public health restrictions throughout the first three waves of the pandemic in Nova Scotia and the province’s relatively low case counts compared to other regions of Canada (and other countries such as Australia and New Zealand), this exploratory sequential mixed-methods study examined the relationship between the COVID-19 pandemic and movement behaviours of children and youth living in Nova Scotia across three time points. To achieve this, survey, regional spatial, and interview data was used to answer the following questions:

1. How did Nova Scotian children and youth’s movement behaviours differ across three time points (i.e., beginning of pandemic, six months into the pandemic, one year into the pandemic) of the COVID-19 pandemic, and are there significant differences across time and between demographic characteristics?
2. Are there geographic concentrations of children and youth in Nova Scotia who were more or less likely to meet the MVPA guideline during the pandemic?
3. What are the perspectives of parents of Nova Scotian children on how movement behaviours changed across the pandemic and how their environment may have helped or hindered their children’s movements, and how do they relate to the above two questions?

## Methods

### Study design

This study used an explanatory sequential mixed-methods design [29]. Quantitative data were first collected through three repeated cross-sectional online surveys and spatial cluster analysis. Qualitative data was also collected through semi-structured interviews. Qualitative data were then analyzed deductively to expand upon quantitative findings. The quantitative parent-reported survey data were used to determine how Nova Scotian children’s and youth’s movement behaviours differed across three time points of the COVID-19 pandemic, if there were significant differences among groups, and if there were geographic concentrations of children and youth in Nova Scotia who were more or less likely to meet the MVPA guideline during the pandemic. The qualitative interviews with parents explored the impact of the pandemic and related health restrictions on their children’s daily movement behaviours and their community resources. This study was approved by Dalhousie University’s Research Ethics Board (#2020-5351, #2022-6040).

### Participants and recruitment

The three nationwide repeated cross-sectional online surveys were disseminated by ParticipACTION (April 2020, October 2020, and April 2021), and conducted by Maru/Matchbox, a third-party market research company. Maru/Matchbox was advised to recruit 1,500 families for each survey with children and youth aged 5 to 17 years, representing Canadians in terms of their child’s age, gender, ethnicity, geographic region, and socioeconomic status. The full recruitment details and survey protocol can be found elsewhere [6]. The repeated cross-sectional design of the study resulted in 291 unique responses provided by parents of children and youth living in Nova Scotia across three surveys (55 (beginning of pandemic), 133 (six months into pandemic), and 103 (one year into pandemic)). Families in Nova Scotia were oversampled in the second and third iterations of the survey to allow for a more robust analysis. For the spatial cluster analysis, all 291 survey responses were considered; however, the survey data from one year into the pandemic included missing residential locations for some children and youth, and as a result, the final sample for this spatial analysis included 228 children and youth. Finally, parents of children who participated in the survey who expressed interest in participating in an interview at the end of the survey were eligible to participate if they met the following inclusion criteria: (a) being a parent or guardian (“caregiver”) of a child ages 5 to 11 years; (b) living in Nova Scotia; (c) able to understand and communicate in English; and (d) having access to a computer with the capability to access Zoom and with a reliable internet connection. A total of 14 caregivers completed interviews.

### Measures and procedures

#### Quantitative

##### Survey

The survey consisted of key content areas developed using the socioecological model to collect information on parental and child demographics, current movement behaviours, changes in children’s and youth’s movement and play behaviours, and ways the child or youth engaged with their environments. The survey also asked for neighbourhood identifiers to characterize the participants’ built and social environments (e.g., six-digit postal codes). Canadian postal codes represent small geographical areas with an average of 19 households per postal code [30]. The variables of interest from the survey for this study included demographic characteristics and each of the three daily movement behaviours, which are the number of days in the last week the participant’s child or youth participated in at least 60 min of moderate-vigorous physical activity (MVPA), and the number of hours per day of both recreational ST and sleep. Light-intensity PA (LPA) was not included as it is difficult to measure and report [31], lacks evidence as to its health benefits [32], and the survey did not have an adequate measures of this PA intensity. Meeting versus not meeting each of the guidelines was based on the Canadian 24-Hour Movement Guidelines for Children and Youth, where children aged 5–17 should accumulate at least 60 min of MVPA per day, spend no greater than two hours in recreational ST, and get 9 to 11 h (ages 5 to 13 years) or 8 to 10 h (ages 14 to 17 years) of quality, uninterrupted sleep [12]. Previous analysis demonstrated that the survey showed strong test–retest reliability [6].

##### Spatial cluster analysis

We conducted spatial analysis to explore the geographic concentration of children and youth more likely to meet the MVPA guideline during the pandemic. We selected to assess MVPA given the likelihood that these behaviours were being influenced by public health restrictions that were inhibiting structured and unstructured PA, such as sport and active play. Due to commonalities in the built and social environments, those living close to each other may demonstrate similar health behaviours, that is, their behaviours may be spatially auto-correlated [33]. A local cluster analysis identifies statistically significant local clusters of desirable versus less desirable outcomes within a regional landscape and can be used to design area-specific policy and health promotion measures. A limited but emerging research has applied spatial analysis methods in examining children’s and youths’ PA outcomes [33–35]. Health geographers have also used this approach in the surveillance and monitoring of disease and health outcomes during the ongoing COVID-19 pandemic [36, 37], although this approach has yet to be applied to examine spatial patterns of children’s health behaviours during the pandemic. For this portion of the analysis, we specifically explored the parent-reported number of days in a week when a child or youth met the daily recommendation of 60 min of MVPA, as well as the residential location of children/youth in our pooled sample using their reported six-digit postal codes from the surveys.

#### Qualitative

##### Semi-structured interviews

The qualitative interviews provided in-depth insights into the caregiver’s perspectives and lived experiences of how their children’s movement behaviours changed across the pandemic and how their environment may have helped or hindered these movements. Qualitative description is an exploratory method that focuses on participants’ lived experiences and emphasizes the participant's voice [38]. Parents of children ages 5 to 11 years living in Nova Scotia participated in semi-structured interviews in June and July of 2021, 15 to 16 months into the COVID-19 pandemic. One-on-one interviews were conducted through a virtual platform (Zoom). The same researcher conducted all interviews which were approximately 60 to 90 min in length. Semi-structured interviews are used in qualitative description to allow the participants to share their experiences [39]. Example questions are in Additional file 1.

#### Data analysis

##### Survey analysis

Statistical analyses were carried out using SPSS v. 27 (IBM Corp., Armonk, N.Y., USA). First, we performed descriptive analyses (means, standard deviation (SD), and proportions) to summarize parent-reported demographic information and child and youth movement behaviours across the total sample, and separately for the three iterations of data collection throughout the pandemic. To assess differences in health behaviours and proportion of participants meeting movement behaviour guidelines by time, age, and gender, chi-square (χ2) tests, independent t-tests, and one-way between-group analysis of variance (ANOVA) tests were used, depending on the variable. Statistical significance was set at *p* < 0.05.

##### Spatial cluster analysis

First, the residential locations of children/youth in our pooled sample (n = 228) were geo-coded and mapped using the six-digit postal codes in ArcGIS Pro®. Second, a global Moran’s I was estimated to examine the presence of spatial auto-correlation in the MVPA data. A statistically significant result (z = 3.85; p < 0.001) indicated the presence of a spatial pattern across Nova Scotia. In other words, the results indicated the presence of local clusters where children/youth who were active (i.e., met the MVPA recommendation) more days of a week were likely to live closer to others who also were active more days of the week, and vice-versa. Next, the Getis-Ord Gi* statistic [40, 41] was estimated using ArcGIS Pro® to identify these local geographic concentrations or MVPA clusters within the study area. We were interested in spatial concentration rather than spatial association. For example, the special case of negative spatial auto-correlation, that would be detectable using local Moran’s I (i.e., zones with high or low values for the variable in question, surrounded by zones with dissimilar values) has not been explored here [33]. The mathematical expression of the Gi* statistic, as employed in our analysis, takes the following form [40]:

$$G_{i}^{*} = \frac{{\sum\limits_{j = 1}^{n} {w_{ij} x_{j} - \overline{x} \sum\limits_{j = 1}^{n} {w_{ij} } } }}{{\frac{{\sqrt[s]{{n\sum\limits_{j = 1}^{n} {w_{ij}^{2} - \left( {\sum\limits_{j = 1}^{n} {w_{ij} } } \right)} }}}}{n - 1}}}$$,$$\forall i \, = \, j$$.

where $x_{j}$ is the number of days in a week (0 to 7) when a child/youth meets recommended MVPA levels,$\overline{x}$ is the mean days for all children/youth (4 days) of meeting the MVPA recommendation across Nova Scotia, $s$ is the standard deviation of the $x_{j}$’s, *n* is the number of observations (n = 228) in the study area, and $w_{ij}$ denotes a binary (i.e., one/zero) spatial weights matrix [40]. We applied a nearest neighbour contiguity rule for the weight matrix, $w_{ij}$ assuming spatial auto-correlation only between a child/youth and 5 “neighbours” from our sample who live closest to them.

Simply put, the local average (i.e., mean number of days of meeting the MVPA recommendation for a child/youth and five closest “neighbours”) is compared proportionally to the average of all observations across the province. When the local mean is statistically different from the “expected” province-level mean, a statistically significant z-score is produced. In this paper, we have grouped the ZGi* scores in three classes: (1) Clusters of high MVPA rates (or more days of meeting MVPA recommendation) with significantly positive ZGi* at α = 0.05, (2) clusters of low MVPA rates (or fewer days of meeting MVPA recommendation) with significantly negative ZGi*, and (3) un-clustered/random observations where ZGi* scores were not statistically significant at α = 0.05.

##### Interview data analysis

After exploring the quantitative findings, we analyzed interview data deductively using reflexive thematic analysis informed by Braun and Clarke [42, 43]. Reflexive thematic analysis is a way to explore and develop themes using qualitative data while reflecting on the researcher’s role [43]. Researchers who were responsible for analyzing the data approached this research with a physical activity and sport science background. More specifically, two white female graduate students living in Nova Scotia transcribed and analyzed the data. Similar to others on the research team, these researchers value physical activity and outdoor play, and experienced their own challenges in engaging in movement during the COVID-19 pandemic. Researchers also live in Nova Scotia and have a personal understanding and interpretations of the impacts of COVID-19 and related restrictions in this province. Researchers who conducted theses analyses did not have first-hand personal experience of being a caregiver to a child during the pandemic or of being a member of a marginalized racial group. As such, analyses and findings were reviewed with other members of the larger author group who had these experiences. Research assistants kept a reflexive journal, reflecting upon their positionality and how their values and beliefs may have shaped their views, broadly and more specifically, regarding this research. Research assistants were provided with transcripts, and they gained familiarity with the data by reading and re-reading the transcripts, taking notes, and reflecting upon them. To integrate the quantitative and qualitative data, codes were developed deductively using the significant quantitative findings from the survey and spatial analysis results. This approach ensures the findings are connected to and informed by the quantitative results, enhancing the overall validity and robustness of the research findings. All coding was completed using NVivo software (Release 1.7.1., QSR International). Collaborative coding occurred at the beginning until familiarity and comfort with the dataset were reached. Following, each research assistant independently coded the remaining transcripts, with the use of a codebook developed amongst research assistants to assist with the coding process. Codes were then examined by a different author to identify patterns of meanings (themes) in light of the quantitative survey and spatial analysis findings. Themes were named collectively by the team after examination and mapping of key quotes in consideration with quantitative findings. Overall, our team thoroughly documented their research activities and processes, carefully described these data and identified themes, and documented contextual factors that may influence these data and our analyses. Analyses were strengthened through the researcher’s positionality, in that they could explore the nuances of the data given their understanding of the quantitative findings and ways COVID-19 had impacted Nova Scotians. Analyses were reviewed and discussed by the larger team before finalizing presentation of qualitative findings. These processes contributed to the study’s quality and rigour (i.e., the care in which the data were collected, analyzed, and interpreted). More specifically, to achieve credibility, the researchers took time and care to examine the data and researchers located themselves in the research with consistent approaches to inquiry [44]. To achieve meaningful coherence, the researchers interpreted the data to tell a coherent story in line and made associations with the quantitative findings [42]. Researchers spent considerable time in the data to develop internally coherent and consistent themes.

## Results

### Participants

A total of 291 parents (mean age = 43.3 years; SD = 9.03) living in Nova Scotia completed the survey at one of the three time points (first, second, and third data collection period: 55, 133, and 103, respectively). Given the purposeful recruitment strategy of the larger Canadian surveys, participants reflected family geographic, ethnic, and socioeconomic distribution and children’s age distributions comparable to Nova Scotia census data [45]. Table 1 shows the descriptive statistics for all Nova Scotian participants and across the three iterations of the survey. Most respondents were women (62.2%), married or common-law (72.9%), had completed college or university (72.5%), and were employed full-time (67.4%). Parents reported the gender of their child using the following options: “boy,” “girl,” “they identify as (describe),” and “I’d rather not say.” Our sample included 49.8% girls, 49.5% boys, and 0.7% respondents who opted not to report gender. The most common dwelling type was a detached home (78.4%). There were no statistical differences in demographics between periods of data collection. Of these 291 participants, 228 were included in the spatial cluster analysis, and 14 caregivers were interviewed.

**Table 1:** Nova Scotian caregiver, child, and youth demographics, and child and youth movement behaviour guideline adherence

Parent demographic profile	Total n = 291	Survey 1 n = 55	Survey 2 n = 133	Survey 3 n = 103
Age, M (SD)	43.32 (9.0)	44.60 (8.3)	41.91 (9.5)	44.45 (8.7)
Gender woman, *n* (%)	181 (62.2)	32 (58.2)	84 (63.2)	65 (63.1)
Ethnicity, *n* (%)				
European	228 (78.4)	52 (94.5)	97 (72.9)	79 (76.7)
Asian	40 (13.7)	1 (1.8)	24 (18.0)	15 (14.6)
Indigenous	10 (3.4)	1 (1.8)	5 (3.8)	4 (3.9)
Other	13 (4.5)	1 (1.8)	7 (5.3)	5 (4.9)
Marital status, *n* (%)				
Married or common-law	212 (72.9)	40 (72.7)	94 (70.7)	78 (75.7)
Divorced or separated	38 (13.1)	10 (18.2)	14 (10.5)	14 (13.6)
Single	39 (13.4)	4 (7.3)	25 (18.8)	10 (9.7)
Widowed	2 (0.7)	1 (1.8)	0 (0)	1 (1.0)
Education, *n* (%)				
High school or less	40 (13.7)	3 (5.5)	23 (17.3)	14 (13.6)
College/Technical	112 (38.5)	21 (38.2)	48 (36.1)	43 (41.7)
University	99 (34.0)	24 (43.6)	41 (30.8)	34 (33.0)
Advanced degree	40 (13.7)	7 (12.7)	21 (15.8)	12 (11.7)
Annual household income, *n* (%)				
< $50,000	63 (21.6)	12 (21.8)	31 (23.3)	20 (19.4)
$50,000 to < $100,000	131 (45.0)	21 (38.2)	62 (46.6)	48 (46.6)
$100,000 +	78 (26.8)	15 (27.3)	32 (24.1)	31 (30.1)
Undisclosed	19 (6.5)	7 (12.7)	8 (6.0)	4 (3.9)
Employment status, *n* (%)				
Full-time (> 30 h/week)	196 (67.4)	44 (80.0)	80 (60.2)	72 (69.9)
Part-time	25 (8.6)	4 (7.3)	16 (12.0)	5 (4.9)
Homemaker	30 (10.3)	3 (5.5)	17 (12.8)	10 (9.7)
Other	40 (13.7)	4 (7.3)	20 (15.0)	16 (15.5)
Child and youth demographic profile				
Age, M (SD) / *n* (%)				
5–11 years	8.16 (2.1) / 137 (47.1)	7.75 (2.0) / 24 (43.6)	7.84 (2.2) / 62 (46.6)	8.75 (1.8) / 51 (49.5)
12–17 years	14.78 (1.7) / 154 (52.9)	14.74 (1.7) / 31 (56.4)	14.45 (1.8) / 71 (53.4)	15.25 (1.5) / 52 (50.5)
Gender, *n* (%)				
Girl	145 (49.8)	31 (56.4)	61 (45.9)	53 (51.5)
Boy	144 (49.5)	24 (43.6)	70 (52.6)	50 (48.5)
I’d rather not say	2 (0.7)	0 (0.0)	2 (1.5)	0 (0.0)
Household makeup, M (SD)				
Adults	2.11 (1.8)	2.00 (0.8)	1.93 (0.6)	2.40 (2.8)
Children	1.66 (0.9)	1.64 (0.7)	1.62 (0.9)	1.73 (0.9)
Child’s residence type, *n* (%)				
House	228 (78.4)	47 (85.5)	101 (75.9)	80 (77.7)
Apartment/Townhouse	59 (20.3)	7 (12.7)	30 (22.6)	22 (21.4)
Other	4 (1.4)	1 (1.8)	2 (1.5)	1 (1.0)
Parent assessed child and youth movement behaviours				
Meeting guidelines, *n* (%)				
Moderate to vigorous physical activity (MVPA)	62 (21.3)	12 (21.8)	29 (21.8)	21 (20.4)
Sleep^1^	189 (64.9)	37 (67.3)	82 (61.7)	70 (68.0)
Screen time	52 (17.9)	2 (3.6)^(2,3)^	26 (19.5)	24 (23.3)
24-h combined	16 (5.5)	1 (1.8)	8 (6.0)	7 (6.8)

Survey 1: beginning of pandemic (April 2020), Survey 2: six months into pandemic (October 2020), Survey 3: one year into pandemic (April 2021). M: Mean; SD: Standard deviation. ^1^Sleep guidelines for different ages (5–13 years: 9–11 h); 14–17 years: 8–10 h) considered. Superscript number(s) identifies a significant difference from another data collection period within the category (p < 0.05), e.g., superscript 2,3 at survey 1 identifies a significant difference between that value and survey 2, and that value and survey 3

### Survey quantitative results

As Table 1 identifies, most Nova Scotian children and youth (64.9%) consistently met sleep recommendations throughout the pandemic, yet far fewer met the MVPA (21.3%) and ST (17.9%) guidelines. The percentage of Nova Scotian children and youth meeting MVPA and sleep guidelines remained stable across data collection periods of the pandemic (April 2020, October 2020, April 2021). Adherence to ST recommendations was significantly lower at the beginning of the pandemic (3.6%) compared to six months and one year into the pandemic (19.5% and 23.3%) (p = 0.011). When looking at meeting all the 24-h movement guidelines (i.e., MVPA, ST, sleep), only 5.5% of children and youth in Nova Scotia were meeting all guidelines throughout the pandemic. This percentage increased as the pandemic went on, from the beginning (1.8%) to six months (6.0%) and one year (6.8%); however, it was not statistically different. As for demographic predictors of meeting the movement behaviour guidelines, chi-square tests found that parents’ education and income each significantly related to adherence. Children and youth of parents reporting a college/technical education were less likely to meet the MVPA guideline compared to children and youth whose parents had an advanced degree (*p* = 0.002). Children and youth whose parents had a university education were more likely to meet sleep guidelines (*p* = 0.042). Children were more likely to meet the 24-h movement guidelines if their parent had an advanced degree (*p* = 0.024). Children and youth whose parents reported an annual household income of $50,000–< $100,000 were less likely to meet the ST (p = 0.029) and MVPA guidelines (*p* = 0.008); an annual household income of > $100,000 corresponded with a greater likelihood of meeting the ST (*p* = 0.029), MVPA (*p* = 0.008), and sleep guidelines (*p* = 0.008). Children and youth whose parents did not specify an annual income were less likely to meet sleep guidelines (*p* = 0.008). A further break down of Nova Scotian participants’ movement behaviours by age group and gender across the three data collection periods was also completed [see Additional files 2, 3].

Although not described in the tables, differences between all children and youth, as well as all girls and boys, were explored. Children obtained more sleep (9.02 vs. 8.54 h/day, *p* = 0.003) and less ST (3.39 vs. 4.53 h/day, *p*≤0.001) than youth, and a higher proportion of children (25.5%) met the ST guidelines than youth (11%) (*p* = 0.002). Only ST guideline adherence differed by gender in the whole sample (31.9% boys, 12.9% girls; *p* ≤ 0.001).

### Spatial cluster analysis results

**Fig. 1:**
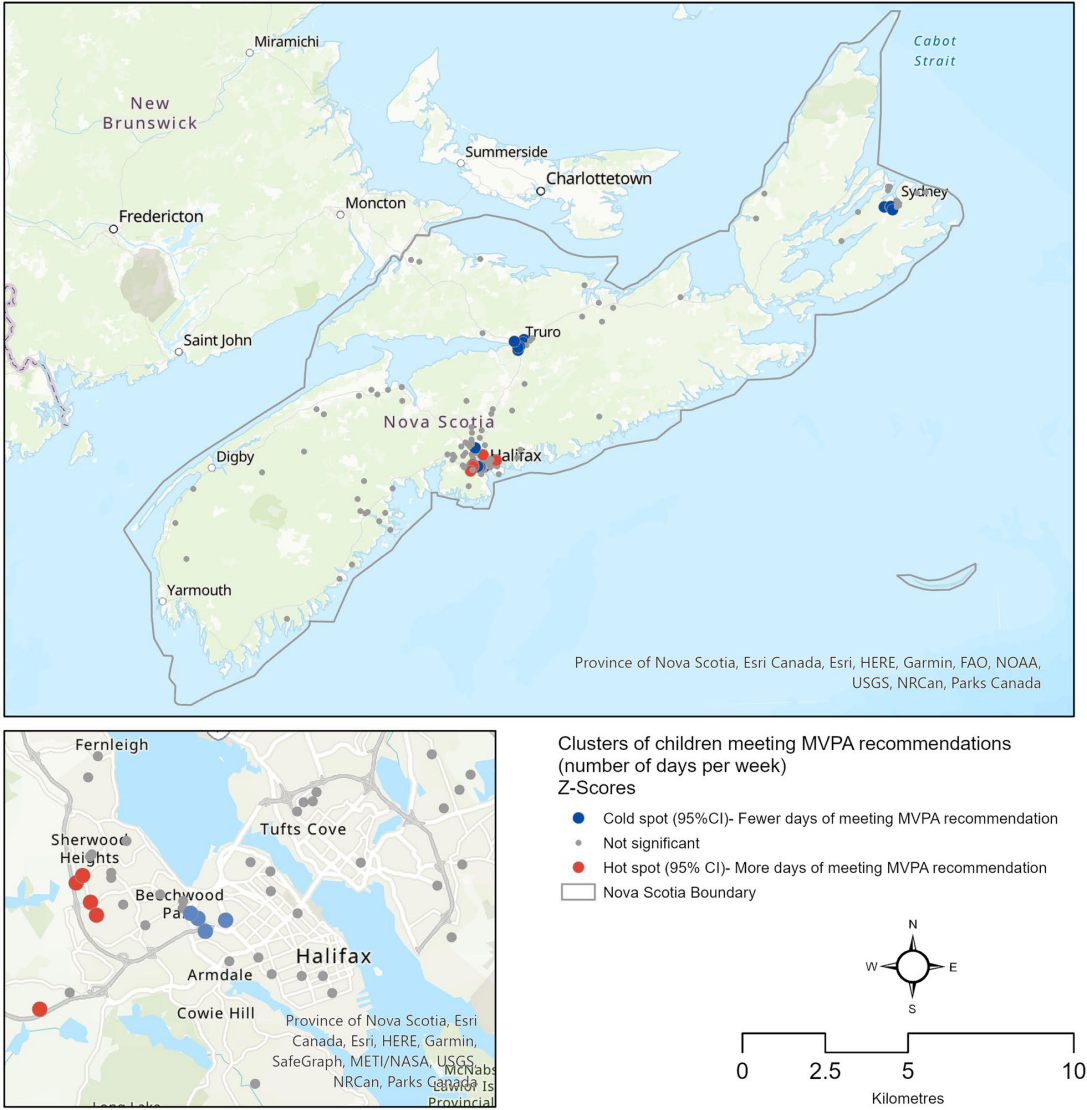
Clusters of children and youths’ residential locations, based on the number of days meeting the MVPA recommendation

ZGi* scores indicated some statistically significant clusters of high MVPA rates (‘hot spots’) and low MVPA rates (‘cold spots’). Figure 1 presents the visual results. At a regional scale, the only systematic concentration of children/youth with high MVPA levels (i.e., cluster of children/youth who were collectively meeting the MVPA for more days than the provincial average of the sample) or ‘hot spot’ is in the Halifax Regional Municipality (HRM) area, which is the largest urban centre within the province. Whereas ‘cold spots’ or concentrations of children/youth with low MVPA levels (i.e., cluster of children/youth who were collectively meeting the MVPA recommendation for fewer days than the provincial average) were found in smaller municipalities such as Truro and Sydney, as well as within the HRM. When taking a closer look, more nuances appear, especially within the HRM. Hot spots were found in the outskirts of Halifax in the Clayton Park West area, which has an average population density of approximately 7318 people/sq. km, many mid- and high-rise housing [46], proximity to an expressway, and less densely connected streets [47]. The cold spots were in the West End of Halifax, which has an average population density of approximately 3645 people/sq. km, a mixture of low-rise/rowhouse and single/duplex dwellings [46], and more densely connected local and arterial streets [47].

### Qualitative findings

Demographic data was collected from 14 caregivers, 10 of which identified as being mothers, 3 identified as being fathers and 1 caregiver identified as a parent but did not specify their gender. The majority of caregivers were married (n = 9), while others reported being single parents (n = 4) or in a relationship (n = 1). Caregivers reported the gender of their child(ren) in the interviews, representing a total of 10 boys and 11 girls.

Qualitative data provided important context to the quantitative results. Three qualitative themes exploring parental perspectives of how Nova Scotian children’s movement behaviours changed across the pandemic and the impact of the child’s environment were developed: (1) escaping screens during early parts of the COVID-19 pandemic and when weather was colder was hard; (2) having access to spaces to be active near the home helped facilitate children’s movement; and (3) higher socioeconomic status enabled more opportunities for movement.

#### *Escaping* screens during early parts of COVID-19 and when weather was colder was hard

This theme highlighted parent-perceived nuances in children’s movement across the pandemic and illustrated changes that may have occurred with seasonal changes. The beginning of the pandemic was notably hard on families, and parents perceived their child’s PA to be lower, particularly when schooling moved online.

One parent described cessation of their child’s pre-programed activities at the start of the pandemic: *“When things were in lockdown, there were less places to go. Even though we had ski passes, we couldn’t leave to ski, that was a barrier. We sign them up for a program, then the program was canceled, that was a barrier”* (parent 4). Another parent described fewer opportunities to play in community spaces or socialize during the early parts of the pandemic: *“It was hard on [child]. [Child] expressed many times that they missed going out… it became a lot of blame COVID – can’t go to the park or see friends, blame COVID”* (parent 12). Parents shared that ST was replacing times of being active during the early parts of the pandemic: *“So, that's pretty much where it started for us was with more TV and video games. Because they were never at sports practices, they were never at friends’ houses, they were never outside riding their bike. It was like a ghost town everywhere around here. I mean… you didn't want to be the person whose kids were outside playing and have people say ‘why are their kids outside playing when they should be inside’. So, that's pretty much what they did in the day”* (parent 7).

As restrictions loosened in the later months of the pandemic (e.g., spring and summer of 2021), parents noted their children returning to play: *“they are back to seeing their friends, they are all allowed out of the field like on school yard and in the parks, they are back to playing you know just catch or football or whatever”* (parent 5). Parents then described a return to *“normal”* in the later months of the pandemic: “*We’ve been able to do a lot more things. We’re able to go outside and play with family and play with the kids in the neighbourhood and [child’s name] can just be a kid and we’re able to go, like I said, to the beach or run around in the woods and explore the trees. Like they’re able to do so many things now as opposed to just a couple months ago and it’s honestly made such a difference”* (parent 2). Winter months were said to be more difficult, with some parents noting fewer opportunities for movement: *“…but when it came to the winter, like I said we got a couple snow storms and they can play in the snow, but other than that we were pretty well locked in because we couldn’t go anywhere”* (parent 2). As the summer of 2021 approached, now 16 months into the pandemic, parents noted less ST and a return to the outdoors: *“More during the summer, it was easier for them to go out because there were other people around. Other people were not on their own electronics playing games either, so [child’s name] wasn’t attracted to that as much because it was not as difficult last summer to go outside”* (parent 1).

Overall, parents noted that the earlier days and months of the pandemic were the most difficult, where the cessation of activities was related to a reduction in PA and an increase in ST. In contrast, later in the pandemic, when restrictions started to loosen, parents described their children returning to activities, to the outdoors, and PA more generally. With this return to what parents called *“normal”* (parent 5, parent 7, parent 9) also came a reduction in children’s ST. While few parents described the weather as a factor determining their child’s movement behaviours, some described the winter months as harder (when more children were indoors and on screens) and the summer months as easier (when more children were outside).

#### Having access to spaces to be active near the home helped facilitate children’s movement

This theme highlighted community and household factors that parents perceived to influence their child’s movement during the pandemic. Parents with children who had safe access to trails, parks, and yards reported that these spaces allowed their child(ren) to continue engaging in outdoor play and PA. Families with less access to these resources (e.g., smaller town, fewer community resources, apartment dwelling) described their location as resulting in less outdoor time and PA for their child. Some parents who lived in more rural areas noted fewer opportunities: *“We live a little bit outside of town so there really is not much around us”* (parent 8). Whereas other parents described their child accessing community spaces: *“We live pretty close to the school that they attend, we live in a pretty quiet area, uh there is a cul de sac just down at the end of the road so lots of… lots of chance for the kids to play outside”* (parent 7) and *“There’s an actual like park with a baseball field in it, field, basketball court, swing, you know that kind of thing, slide, nice area, trails to walk around, that sort of stuff too”* (parent 15).

The family’s dwelling type was also relevant. Families who lived in apartments noted fewer opportunities to be outdoors and for PA: *“And, um, we are in an apartment building, so it has been a little confining”* and *“in the dead of winter when you are not getting enough sunlight as it is and you are kind of trapped and being in an apartment building, it is [even worse]. You don't have the freedom to move out and have your own space"* (parent 5). Families who lived on busier streets noted feeling less comfortable with their child biking: *“I am not really comfortable … letting him go when it is busy because of traffic and that he is 11 and I don’t trust that [child’s name] is paying attention”* (parent 1). Families who lived in a home with a yard described more opportunities for outdoor play: *“We have a playground in the backyard. [Child’s name] plays street hockey in the yard”* (parent 1). Parents described their comfort in having their child play outside in the yard: *“I think it definitely got easier …we were still comfortable with our kids being outside, we were still comfortable with them playing in the yard … they were able to play more in the yard with either us or we would go outside with our dogs”* (parent 2).

Overall, this theme suggests that children who had access to spaces at their home (e.g., backyard) or within the community (e.g., playground, school ground) were afforded more opportunities to be outside and engage in PA within the confines of pandemic restrictions.

#### Higher socioeconomic status enabled more opportunities for movement

This theme brought to light additional inequities children faced because of their socioeconomic status. Children of families with higher household incomes described more opportunities to engage in play, sometimes due to larger properties, recreation-related purchases, or vacation opportunities. For example, one family described adding a basketball court to their yard during the pandemic: *“What we ended up doing was we put concrete slab in the backyard and made a basketball court for them”* (parent 4). The same family added a pool to their yard during the pandemic: *“Yeah, we have a big back yard and this year we actually put in a pool anticipating that we were not going anywhere, and they were not going to be in a lot of camps”* (parent 4). Families who could go camping with their child(ren) also noted benefits to their child’s movement, more PA and less ST: *“When we go camping, we take out bikes and we hike. If it is warm, we go swimming. There is less electronic use at that time, but it is because it is not available mostly”* (parent 1). This included children who were enrolled in camps: *“For this week, they are in a camp, so their screen time is down”* (parent 5). Living on or near the water also enabled children’s movement: *“We were blessed living on a lake and everyday going swimming. They have a water trampoline. They get their exercise when they are out on the water – that was good”* (parent 10).

Overall, this theme suggests that some families had greater means to accommodate movement during stay-at-home orders, e.g., able to make significant purchases by installing sports courts or pools. Like the previous theme, those with more space, including access to trails, parks, or lakes, described engaging in the outdoors during the pandemic. Many parents noted opportunities for vacations or to escape to their cottage for pandemic respite. This theme indicated that although pandemic-related restrictions were similar across Nova Scotia, some families were able to experience less disruption to their movement due to higher socioeconomic status.

## Discussion

This explanatory sequential mixed-methods study aimed to summarize the collective findings from the survey, regional spatial analysis, and analyzed in-depth interviews to help illustrate the impact of the COVID-19 pandemic on Nova Scotian children’s and youth’s movement behaviours. Nova Scotia is one of the less populated provinces in Canada; however, it still has a population of just over one million residents. Approximately half of those residents live in HRM, with other medium- and small-sized communities comprising the rest of the province. Given that Nova Scotia experienced varying levels of COVID-19 cases and related public health strategies to mitigate the spread of the virus at the three different study time points has not been studied on its own in previous analyses, and provides an interesting geographic lens in how its relatively isolated nature allowed it to mitigate virus transmission, it was important to assess its relationship to children and youth’s healthy movement behaviours such as MVPA, sleep, and ST. By investigating aspects of the SEM that have shown to influence movement, such as regional differences in movement behaviours, specifically MVPA, during the pandemic, we have provided evidence for potential protective or harmful environmental and sociodemographic factors that are related to healthy movement behaviours and can provide guidance for future environmental interventions. Further, because children and youth in Atlantic Canada had some of the best movement behaviours adherence in Canada throughout the first six months of the pandemic [1, 3], using the combination of three surveys (including one year into the pandemic), environmental data, and interviews of parent perceptions provided the opportunity to explore how the pandemic progressed and affected the movement of children and youth living in Nova Scotia.

The survey findings demonstrate that, similar to national data [6, 7], very few Nova Scotian children and youth were meeting movement behaviour guidelines during the pandemic. Nationally, just 4.8% of children (2.8% girls, 6.5% boys) and 0.6% of youth (0.8% girls, 0.5% boys) met combined 24-h movement guidelines (PA, ST, sleep) during the early stages of the pandemic (April 2020); guideline adherence was similarly low six months later (October 2020). In Nova Scotia, 5.5% of children and youth met the combined movement behaviour recommendations across the pandemic; guideline adherence appeared to increase slightly from the beginning of the pandemic (April 2020) to six months (October 2020), then remaining stable one year into the pandemic (April 2021), although not significantly different across time. The results indicate that Nova Scotia had higher adherence to the combined guidelines than Canada as a whole. Similar to the findings from regional breakdowns, lower case counts likely influenced these trends compared to larger Canadian provinces. Thus, initially closing its boarders may have contributed to fewer cases, allowing less strict public health guidelines, such as students returning to school and recreation spaces re-opening earlier, may have allowed for greater opportunities for favourable movement behaviours [1]. Despite the higher proportions of guideline adherence in Nova Scotia, these results are concerning given already low pre-pandemic levels of guideline adherence, with just 17.5% of Canadian children and youth meeting the combined 24-h movement behaviour guidelines from 2009 to 2013 [48]. Consequently, as we adapt to living in the era of COVID-19, there is a need to strive to not only return to these levels but exceed them.

When breaking down the survey findings by specific behaviours, ST was found to be related to the most significant differences across time. ST was significantly elevated throughout the beginning of the pandemic. This was echoed again by caregivers during the qualitative interviews. While restrictions played a part in declining opportunities for PA and outdoor play, the use of screens for children became essential to their education, social interactions, entertainment, and distraction as their parents or caregivers worked from home [49]. This was similarly explained by parents in interviews, such that ST replaced usual in-person social time or became something to do instead of sports practices or playing outside. Because screens became so pervasive in most children’s lives during these times, especially at the beginning of the pandemic, this may have led to it being the most significantly affected movement behaviour for Nova Scotian children and youth.

From the spatial analysis, location within the region may have been associated with children’s MVPA. Interestingly, the only ‘hot spots’ in the province, which indicate statistically significant clusters of high MVPA rates among participants in a certain area, happen to be in the largest municipality, the HRM. These clusters may have been more likely to be detected based on a greater proportion of the province’s survey participants living in the HRM, but the sample is representative of the broader Nova Scotia population. Still, when compared to other parts of the province, ‘cold spots’ were found in other, smaller municipalities such as Sydney or Truro, but also within the HRM.

When looking closer at where the hot and cold spots appeared in the HRM, some patterns emerged. Because this analysis only aims at assessing the regional level, it is difficult to infer much about the detailed neighbourhood-level characteristics of these clusters. Some of the built environment characteristics, such as dwelling type and street connectivity of the hot and cold spots within the HRM are contradictory to other literature and our qualitative findings, suggesting that living in apartments or densely populated areas was detrimental to children’s PA levels during the pandemic [5] or street connectivity being positively related to children and youth’s PA [50]. However, more context may be available when inspecting socioeconomic type data from 2016 census data maps. The areas where cold spots emerged in the West side of Halifax coincide with an area that has about 40% households with lower income levels and 13% unsuitable housing compared to the hot spot area that has less than 15% of households with lower income levels and less than 10% unsuitable housing [46]. Another study of the wider Canadian national-level survey found that compared to those with an annual family income of $35 K to $75 K, children from families earning $75 K to $150 K were more likely to engage in increased outdoor activities throughout the COVID-19 pandemic [5], and this was also the case in this Nova Scotian sample, where participants with an annual household income of $50,000 to < $100,000 were less likely to meet the ST and MVPA guidelines, and > $100,000 annual household income more strongly predicted meeting the three movement behaviour guidelines. This is also highlighted in the interview findings that describe how families with higher SES were able to implement spaces for movement on their properties or to take vacations outside the city near beaches, trails, and lakes, thus supporting movement throughout the pandemic.

People living in low-income communities have been shown to be disproportionately affected by COVID-19. This is due to a plethora of inequities, such as being more likely to have pre-existing conditions leading to a higher risk of severe illness from COVID-19, having less access to medical treatment and insurance, being more likely to work for essential business industries, and living in crowded and multigenerational households with less capacity for isolation [51]. Therefore, children and youth living in these areas in Nova Scotia *may* have experienced more illness, stress, or less support for and modelling of PA within their families. In the national, cross-sectional data, parental support was noted as a facilitator of children’s and youth’s movement behaviours during the pandemic [7]; other parental characteristics associated with adherence to the recommendations included high parental-perceived capacity to restrict ST, with low parental-perceived capability to support children’s and youth’s sleep and PA being associated with non-adherence [4]. Similarly, in the other cold spots in Sydney and Truro, these smaller urban areas have a relatively higher percentage of low-income households than their surrounding areas [46]. Additionally, those living in the hot spot areas are slightly more removed from the more urban inner-city of Halifax. The surrounding areas boast numerous greenspaces, recreation centres, and school yards; therefore, potentially creating more opportunities for children, youth, and their families to access PA and recreational areas during the pandemic.

Although our analyses do not examine community or neighbourhood factors associated with movement behaviours, these environments support movement [5]. In order to combat the unfavourable movement behaviours highlighted throughout the pandemic, the Province of Nova Scotia has worked to support active transport and outdoor play opportunities. For instance, in a study comparing street reallocation in three mid-sized cities in Canada, Halifax reallocated 17.2 km of street space, which was the most of the three cities [52]. These changes included sidewalk expansions, closure of residential streets to non-local traffic, and closure of streets or lanes to create temporary patios and promote social distancing [53]. These modifications allowed for more active transportation, outdoor active play, and a greater sense of safety when using these areas [54]. Decisions and relatively quick action on environmental changes such as these may have contributed to maintaining children and youth’s MVPA levels throughout the first year of the pandemic. Despite much effort, it appears that the impacts on health behaviours have been unequal across the region, highlighting the importance of examining nuances in urban and social conditions and creating policy and programs that are strategically focused on local challenges and needs.

In our qualitative analyses, parents noted that the pandemic stage and time of year influenced their child’s PA and ST. These results were also reflected in the survey, where early in the pandemic fewer children and youth living in Nova Scotia were meeting the movement behaviour guidelines, and in particular, PA was notably down early in the pandemic with a slight rebound in later parts of the pandemic, though still not returning to pre-pandemic levels, as perceived by their parents. When PA was lowest, recreational ST was reportedly at its highest. As children and youth returned to school and recreational activities in the later stages of the pandemic, ST started to return to pre-pandemic levels.

As it related to the spatial analyses, parents described the impact of location, environment, and spaces on their child’s movement behaviours. Some parents suggested that living in rural places or having the space to play outside at home allowed for their child to have more space or access to lakes and forested areas, which was positively related to more PA opportunities. Other parents stated the need to stay close to home or felt limited due to their dwelling type (e.g., living in an apartment), and as a result, the child’s immediate neighbourhood surroundings became their place to play. Parents of children who lived in more densely populated or urban settings noted concerns about nearby traffic and limited their child’s time outdoors. A study of children ages 1 to 5 years in the early stages of the pandemic in Chile also found that children living in rural places or in homes with space to play experienced less decline in their PA levels [55]. These findings somewhat relate to the spatial analyses conducted, for example, West Halifax as a ‘cold spot’ is less densely populated but includes more densely connected streets which could contribute to traffic levels. However, the Clayton Park West area as a ‘hot spot’ includes many apartment buildings contributing to greater population density but may have more access to recreation facilities and schoolyards contributing to more movement. That said, more rural environments were not found to be ‘hot spots’ either, despite some parents reporting more acreage resulted in more outdoor play. In our case, there may not have been enough participants in the rural locations to detect meaningful clusters of meeting the MVPA guideline, whereas interview data showcased some of the advantages to living in these spaces.

Finally, the pandemic and related public health restrictions were not experienced equally. Some parents noted creating spaces for play in their home, investing in sports courts and pools. Other parents described opportunities for respite to the cottage or to take vacations. In the spatial analysis, we also found that affluent areas were ‘hot spots’ and children and youth were more likely to meet the MVPA recommendation in these areas. As noted in the survey findings, income was found to be a predictor of meeting the movement behaviour guidelines, such that those with lower incomes would be less likely to have the means to create these spaces or have access to many areas that promote PA. These findings further emphasize the importance of locally catered policies and programs to address the needs of each community.

This paper includes strengths such as the use of explanatory sequential mixed-methods within a geographic case study to help address the questions using various perspectives and data sources. Being able to integrate the quantitative and qualitative data using deductive analysis provides the richness needed to examine the movement behaviours and related experiences of Nova Scotian children and youth throughout the unique time of the COVID-19 pandemic, at different scales and with various levels of detail. Additionally, this study used data from three different time points of the pandemic, of which the data one year into the pandemic has not been used in the assessment of Canadian regional data to date. Further, a population-representative sample and the same methodological approach was used in each iteration of the study. Despite these efforts, the study has some limitations. First, the study employed a repeated cross-sectional design with different participants in each iteration of the survey; therefore, the results do not directly show changes in movement behaviours throughout the pandemic. However, qualitative interviews address this limitation by providing in-depth accounts of parent-perceived movement behaviours across the pandemic. Second, the sample size for the survey was small for Nova Scotia, especially in the first iteration of data collection. Third, the survey was parent-reported, possibly introducing desirability or recall bias, as well as the parents not being able to report on the child’s behaviours as accurately as the child themselves may be able to. Fourth, PA is broken into different intensities, including LPA which makes up the largest portion of PA in a day and is beneficially associated with health outcomes [56, 57]. However, due to the constraints of reporting LPA behaviours, we only examined MVPA, which accounts for < 5% of children’s typical days [58]. Finally, the interview data only included parents of children, not youth, and did not interview the children and youth themselves, which potentially limits the findings of this paper.

## Conclusions

The pandemic was associated with significant collateral consequences and the disruption of movement behaviours for children and youth living in Nova Scotia, albeit less so than in other regions in Canada and around the world. Routines and schedules were thwarted, contributing to higher ST levels earlier in the pandemic, and children in families of lower socioeconomic status were potentially restricted in PA opportunities. The province implemented strict virus mitigation strategies that allowed more lenience in restrictions, while also showing adaptation and resilience. However, new ways of getting moving beyond structured and recreational activities and maintaining and developing access to spaces to engage in PA should continue to be explored as health promotion strategies. Fortunately, the province has committed to, and taken important steps to, help facilitate this comeback by improving play- and PA-friendly spaces and access to the outdoors. Although small interventions have been implemented, there is still more to do to ensure all children and youth have the opportunity to not only get back to pre-pandemic movement behaviours but to exceed these levels for better health outcomes. Moving forward, children, youth, and their families will need to reimagine what their movement behaviours look like. The pandemic has emphasized the importance of many levels of influence of the SEM, including our immediate surroundings and the need for neighbourhoods and communities to make PA accessible, equitable, and sustainable for all [59]. Ideally, these changes and implementations need to be rectified before the next public health crisis.

## Supplementary Information

**Additional file 1.** Interview Guide (abbreviated).

**Additional file 2.** Nova Scotian children’s movement behaviours across the pandemic.

**Additional file 3.** Nova Scotian youth's movement behaviours across the pandemic.

## Data Availability

The quantitative data for this study are available upon responsible request to the corresponding author and upon the signing of a data transfer agreement. The qualitative data are not publicly available due to concerns that may compromise participant confidentiality and consent.
