# Supplementary material for: Children and youth’s movement behaviours differed across phases and by geographic region throughout the COVID-19 pandemic in Nova Scotia, Canada: an explanatory sequential mixed-methods study
Source: J Act Sedentary Sleep Behav. 2023 Nov 3;2:25. doi: 10.1186/s44167-023-00032-6 (PMC11960356; doi:10.1186/s44167-023-00032-6)
Supplement: Supplementary file 1 — Additional file 1. Interview Guide (abbreviated). [file 44167_2023_32_MOESM1_ESM.docx]

**Additional file 1. Interview Guide (Abbreviated)**

***The World Health Organization (WHO) declared COVID-19 as a pandemic last year, on March 11, 2020. For the following questions, we would like you to consider how your child’s movement behaviours (physical activity, play, sedentary behaviours, screen use, and sleep) changed over the course of the last year as a result of COVID-19 and related restrictions.***

**Introductions and re-confirmation of consent**

**Setting the context**

- Can you describe the current pandemic-related restrictions your family is experiencing where you live? Prompts: what are you allowed to do; what are you not allowed to do?

1. Looking back since March 2020, can you describe what the restrictions were when they felt most challenging? Prompts; what was different from now; how have things changed over time?

**Changes in movement behaviours**

1. Let’s think about a typical day when you felt the restrictions were most challenging. Can you walk me through what that day was like for your child? *Prompts:*
   - outdoor play/time, unstructured PA, play inside home, changes in your permissions? (where/what)
   - independent/unsupervised or with family?
   - Structured PA; Stopped? Modified? Anything new?
   - Active transport
   - Sleep duration/quality
2. How does this compare with a typical day now? *Prompts:*

- outdoor play/time, unstructured PA, play inside home; changes in your permissions? (where/what)
- independent/unsupervised or with family?
- Structured PA; Stopped? Modified? Anything new?
- Active transport
- Sleep duration/quality

1. Looking back, how much has your child’s movement and play behaviours changed between when the time restrictions were most challenging, and today? *Prompts:* screen time, changes when school was in person vs. home learning? Summer/winter?

**Family Behaviours and Changes in Support Behaviours**

1. Comparing pre-pandemic to today, have there been changes in how you support your child(ren) in engaging in healthy movement behaviours? *Prompts*:
   - Encouragement? (play, PA, sleep)
   - Co-participation? (play, PA)
   - Setting screen restrictions?
   - Providing logistic support (e.g., drive to practices)
   - Looking for PA programs to enroll my child in
   - Buying PA/sports equipment to use
   - Have your own movement behaviours changed?
2. What barriers have you faced in supporting your child(ren) to engage in healthy movement behaviours? How have you managed these? *Prompts:* how did these barriers change over the course of the pandemic, are they still present, how have you worked through these barriers? What would (have) help(ed) in facing these?
   - *Job/school requirements*
   - *Neighbourhood environment*
   - *Particular restrictions*
   - *Screen time*
   - *Family factors*
3. To what extent is PA an important part of your family time together right now? How does that compare with the most challenging point of the pandemic? Pre-pandemic? *Prompts*: More or less? Are you doing anything new? Stopped anything?
4. Can you describe what types of discussions about physical activity you currently have with your child? Limiting screen time? *Prompts*:
   - encouragement, benefits, reflection/progress, goals, teaching, praising, rewards
   - Have these conversations changed since the most difficult point of lockdowns? How does this compare with before the pandemic?
5. How insistent would you say you are that your child is physically active right now? Limiting screen time? *Prompts*:

- reminders, nagging, reward/punishment, threaten, guilt
- force them to be active?
- Has your level of insistence/strategies changed since the most challenging point? What about before the pandemic?

1. Can you walk me through how decisions about your child(ren)’s free time are made at this point? *Prompts*: what/when/where, how much say from your child(ren)? How are disagreements handled? Is this different than at the hardest point? Pre pandemic?

**Looking Ahead**

1. Having experienced the impact of physical distancing and other related restrictions, is there anything that would have helped you in supporting your child(ren)’s movement behaviours? *Prompts:* Anything that wished you had known; any resources that you wished you had?
2. What community resources did you access during the pandemic and what community resources would you like going forward as we recover from this pandemic? *Prompts:* have you accessed in person v virtual programs, recreation and park spaces, health services? How have you used community spaces, your neighbourhood environment?
3. This pandemic has been long lasting, but we are starting to see a light at the end of the tunnel. How will things change for your child in terms of their outdoor play, physical activity and screen time over the next six months? *Prompts:* any concerns, when do you expect to return to ‘normal’, what will this new ‘normal’ look like for your family in how you engage with movement and play?
4. How will things change regarding your parenting practices in supporting your child to engage in healthy movement behaviours? Prompts: do you have any plans to make changes in how you support your child?
5. We all recognize how challenging this pandemic has been for kids and their families. Are there any good things that have come out for your child or family during the pandemic? *Prompts:* connecting with others, reimaging leisure, redefining movement, stress coping strategies?
6. Do you have any **advice for *other* families** like yours trying to achieve a healthy balance of movement behaviours (physical activity, play, screen time, sleep) of their children during the ongoing recovery from the COVID-19 pandemic and related restrictions?

**Conclusion**

- Ask for interest in receiving a report of study findings.
- Thank for participation
