# Supplementary material for: Children and youth’s movement behaviours differed across phases and by geographic region throughout the COVID-19 pandemic in Nova Scotia, Canada: an explanatory sequential mixed-methods study
Source: J Act Sedentary Sleep Behav. 2023 Nov 3;2:25. doi: 10.1186/s44167-023-00032-6 (PMC11960356; doi:10.1186/s44167-023-00032-6)
Supplement: Supplementary file 2 — Additional file 2. Nova Scotian children’s movement behaviours across the pandemic. [file 44167_2023_32_MOESM2_ESM.docx]

**Additional file 2. Nova Scotian children’s movement behaviours across the pandemic (n=137).**

|  | **Children (ages 5-11)** | | | | | | | | | | | |
| --- | --- | --- | --- | --- | --- | --- | --- | --- | --- | --- | --- | --- |
|  | **Total** | | | | **Girls** | | | | **Boys** | | | |
|  | **Total (*n* = 137)** | **S1 (n = 24)** | **S2 (n = 62)** | **S3 (n = 51)** | **Total (n = 61)** | **S1 (n = 12)** | **S2 (n = 25)** | **S3 (n = 24)** | **Total (n = 75)** | **S1 (n = 12)** | **S2 (n = 36)** | **S3 (n = 27)** |
| **Movement behaviours, M (SD)** | | | | |  |  |  |  |  |  |  |  |
| MVPA ≥60 min (days/week) | 3.86 (2.17) | 3.46 (2.19) | 3.58 (2.20) | 4.39 (2.05) | 4.11 (2.18) | 4.08 (2.11) | 3.88 (2.37) | 4.37 (2.06) | 3.71 (2.12) | 2.83 (2.17) | 3.47 (2.04) | 4.41 (2.08) |
| Sleep (hours/day) | 9.02 (1.26) | 9.26 (1.18) | 8.75 (1.37) | 9.22 (1.14) | 9.21 (1.12) | 9.42 (1.24) | 9.23 (1.07) | 9.08 (1.14) | 8.88 (1.35) | 9.09 (1.14) | 8.44**^3^** (1.46) | 9.33 (1.14) |
| Screen time (hours/day) | 3.39 (2.57) | 4.94**^2,3^** (3.03) | 2.90 (2.02) | 3.23 (2.64) | 3.73 (2.57) | 5.52 (3.68) | 3.03 (2.38) | 3.59 (1.94) | 3.11 (2.56) | 4.41 (2.38) | 2.82 (1.78) | 2.92 (3.14) |
| **Proportion of children meeting guidelines (%)** | | | | |  |  |  |  |  |  |  |  |
| MVPA | 24.1 | 16.7 | 21 | 31.4 | 29.5 | 25 | 28 | 33.3 | 20 | 8.3 | 16.7 | 29.6 |
| Sleep | 62.8 | 66.7 | 54.8 | 70.6 | 63.9 | 66.7 | 64 | 62.5 | 62.7 | 66.7 | 50 | 77.8 |
| Screen time | 25.5 | 4.2**^2,3^** | 27.4 | 33.3 | 13.1**^‡^** | 0 | 20 | 12.5 | 36^‡^ | 8.3 | 33.3 | 51.9 |
| 24 h combined | 7.3 | 0 | 6.5 | 11.8 | 4.9 | 0 | 8 | 4.2 | 9.3 | 0 | 5.6 | 18.5 |

S: Survey (S1: beginning of pandemic (April 2020), S2: six months into pandemic (October 2020), S3: one year into pandemic (April 2021)); M: Mean; SD: Standard deviation. Superscript number(s) identifies a significant difference from another data collection period within the category (p < 0.05), e.g., superscript 2,3 at S1 identifies a significant difference between that value and S2, and that value and S3. ^‡^ identifies a significant difference by gender (p < 0.05).
