# Supplementary material for: Children and youth’s movement behaviours differed across phases and by geographic region throughout the COVID-19 pandemic in Nova Scotia, Canada: an explanatory sequential mixed-methods study
Source: J Act Sedentary Sleep Behav. 2023 Nov 3;2:25. doi: 10.1186/s44167-023-00032-6 (PMC11960356; doi:10.1186/s44167-023-00032-6)
Supplement: Supplementary file 3 — Additional file 3. Nova Scotian youth's movement behaviours across the pandemic. [file 44167_2023_32_MOESM3_ESM.docx]

**Additional file 3. Nova Scotian youth’s movement behaviours across the pandemic (n=154).**

|  | **Youth (ages 12-17)** | | | | | | | | | | | |
| --- | --- | --- | --- | --- | --- | --- | --- | --- | --- | --- | --- | --- |
|  | **Total** | | | | **Girls** | | | | **Boys** | | | |
|  | **Total (*n* = 154)** | **S1 (n = 31)** | **S2 (n = 71)** | **S3 (n = 52)** | **Total (n = 84)** | **S1 (n = 19)** | **S2 (n = 36)** | **S3 (n = 29)** | **Total (n = 69)** | **S1 (n = 12)** | **S2 (n = 34)** | **S3 (n = 23)** |
| **Movement behaviours, M (SD)** | | | | |  |  |  |  |  |  |  |  |
| MVPA ≥60 min (days/week) | 3.38 (2.23) | 3.61 (2.43) | 3.55 (2.24) | 3.02 (3.09) | 3.39 (2.29) | 4.05 (2.46) | 3.50 (2.21) | 2.83 (2.22) | 3.42 (2.15) | 2.92 (2.31) | 3.71 (2.25) | 3.26 (1.94) |
| Sleep (hours/day) | 8.54 (1.37) | 8.93 (1.73) | 8.37 (1.08) | 8.54 (1.45) | 8.60 (1.26) | 8.83 (1.04) | 8.33 (1.05) | 8.76 (1.55) | 8.47 (1.50) | 9.09 (2.55) | 8.41 (1.13) | 8.26 (1.29) |
| Screen time (hours/day) | 4.53 (2.67) | 5.81**^2^** (2.64) | 4.08 (2.04) | 4.42 (3.06) | 4.68 (2.47) | 5.44 (2.13) | 4.16 (1.90) | 4.77 (3.03) | 4.31 (2.95) | 6.81 (3.74) | 3.96 (2.25) | 3.97 (3.11) |
| **Proportion of children meeting guidelines (%)** | | | | |  |  |  |  |  |  |  |  |
| MVPA | 18.8 | 25.8 | 22.5 | 9.6 | 20.2 | 31.6 | 22.2 | 10.3 | 17.4 | 16.7 | 23.5 | 8.7 |
| Sleep | 66.9 | 67.7 | 67.6 | 65.4 | 65.5 | 68.4 | 66.7 | 62.1 | 69.6 | 66.7 | 70.6 | 69.6 |
| Screen time | 11 | 3.2 | 12.7 | 13.5 | 9.5 | 5.3 | 11.1 | 10.3 | 13 | 0 | 14.7 | 17.4 |
| 24 h combined | 3.9 | 3.2 | 5.6 | 1.9 | 3.6 | 5.3 | 5.6 | 0 | 4.3 | 0 | 5.9 | 4.3 |

S: Survey (S1: beginning of pandemic (April 2020), S2: six months into pandemic (October 2020), S3: one year into pandemic (April 2021)); M: Mean; SD: Standard deviation. Superscript number(s) identifies a significant difference from another wave within the category (p < 0.05), e.g., superscript 2 at S1 identifies a significant difference between that value and S2.
